# Supplementary material for: Homocysteine directly interacts and activates the angiotensin II type I receptor to aggravate vascular injury
Source: Nat Commun. 2018 Jan 2;9:11. doi: 10.1038/s41467-017-02401-7 (PMC5750214; doi:10.1038/s41467-017-02401-7)
Supplement: Supplementary file 1 — Supplementary Information [file 41467_2017_2401_MOESM1_ESM.pdf]

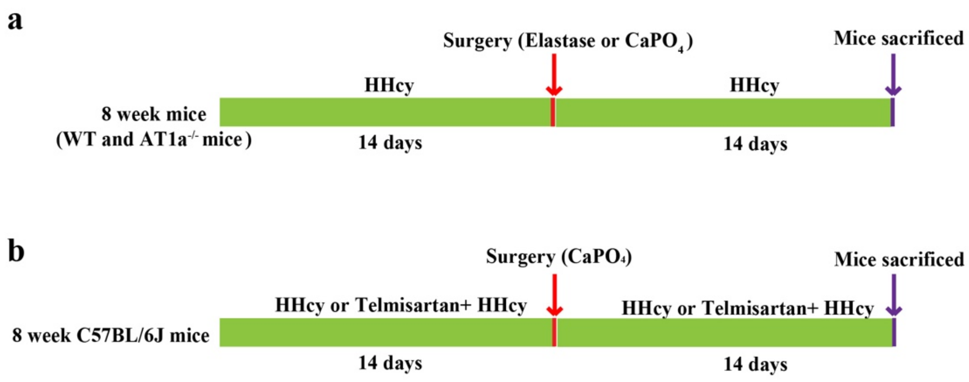

Supplementary Figure 1. Schematic flowcharts of animal models. a, Schematic flowchart of HHcy induction and elastase or $CaPO_4$ treatment in Figure 1a-d and Supplementary Figure 2a-c. **b,** Schematic flowchart of HHcy induction, telmisartan treatment and $CaPO_4$ treatment in Supplementary Figure 3a-c.

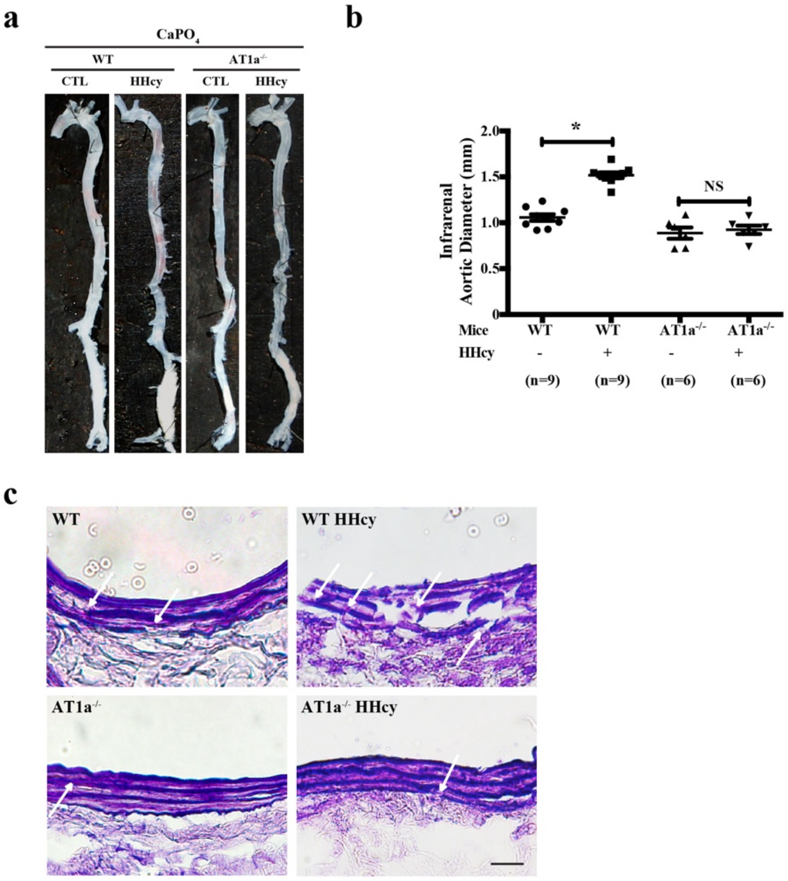

Supplementary Figure 2. HHcy aggravates $CaPO_4$-induced AAA in WT but not *AT1a*$^{-/-}$ mice. a, Representative photographs of $CaPO_4$-induced AAA in WT and AT1a$^{-/-}$ mice, N=6-9. **b,** The quantification of the infrarenal abdominal aortic diameter in mice with $CaPO_4$-induced aneurysms. N=6-9. **P*<0.05. Kruskal-Wallis test followed by Dunn's test. **c,** Representative Gomori staining of elastin degradation. Scale bar, 20 μm, N=6-9.

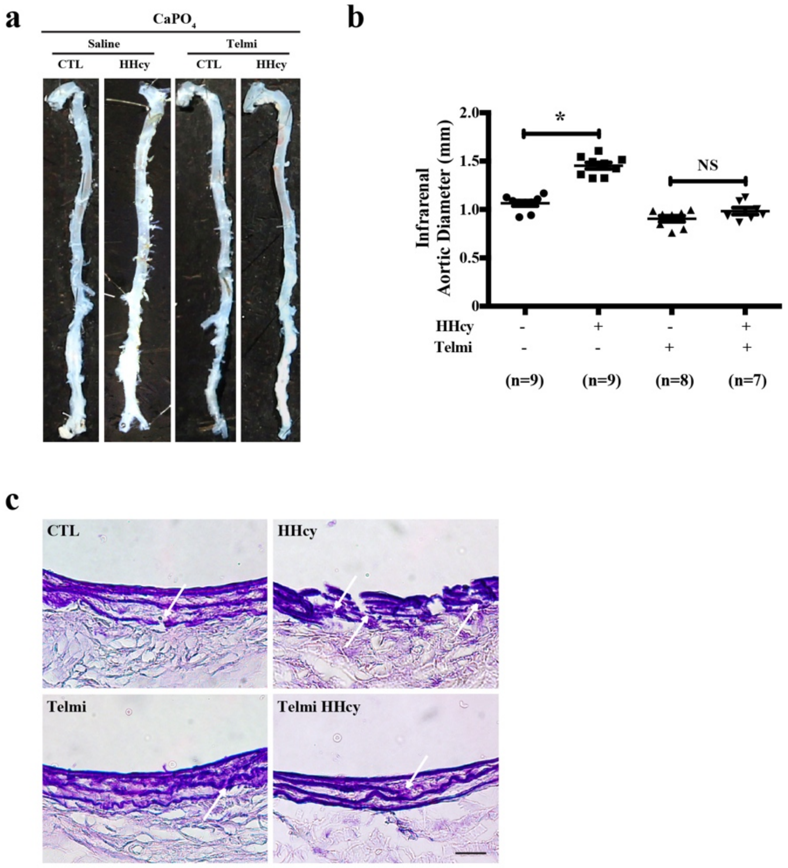

Supplementary Figure 3. Telmisartan treatment rescues HHcy-aggravated AAA in mice. **a,** Representative photographs of $CaPO_4$-induced AAA in C57BL/6J mice fed with or without telmisartan and Hcy. Control (CTL), $CaPO_4$ treatment; HHcy, Hcy (1.8 g/L) in drinking water plus $CaPO_4$ treatment; Telmi, telmisartan in drinking water plus $CaPO_4$ treatment; Telmi + HHcy, telmisartan and Hcy in drinking water plus $CaPO_4$ treatment, N=7-9. **b,** The quantification of the abdominal diameter in mice with $CaPO_4$-induced aneurysms. N=7-9, **P*<0.05, Kruskal-Wallis test followed by Dunn's test. **c,** Representative Gomori staining of elastin degradation. Scale bar, 20 μm, N=7-9.

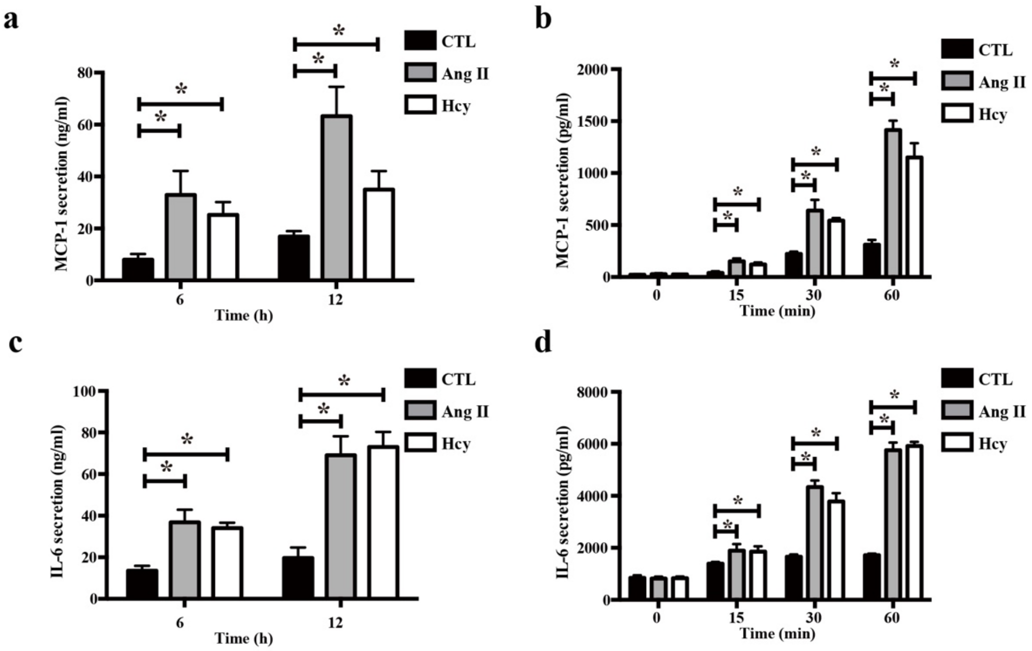

Supplementary Figure 4. Hcy induces MCP-1 and IL-6 secretion. a-b, Ang II (1 μM)- or Hcy (100 μM)-induced C57BL/6J mice abdominal aortic ring MCP-1 secretion at different time points. The data represent as mean ± SEM, N=6, **P*<0.05, One-way ANOVA followed by the Bonferroni post hoc test. **c-d,** Ang II (1 μM)- or Hcy (100 μM)-induced C57BL/6J mice abdominal aortic ring IL-6 secretion at different time points. The data represent as mean ± SEM, N=6, **P*<0.05, One-way ANOVA followed by the Bonferroni post hoc test.

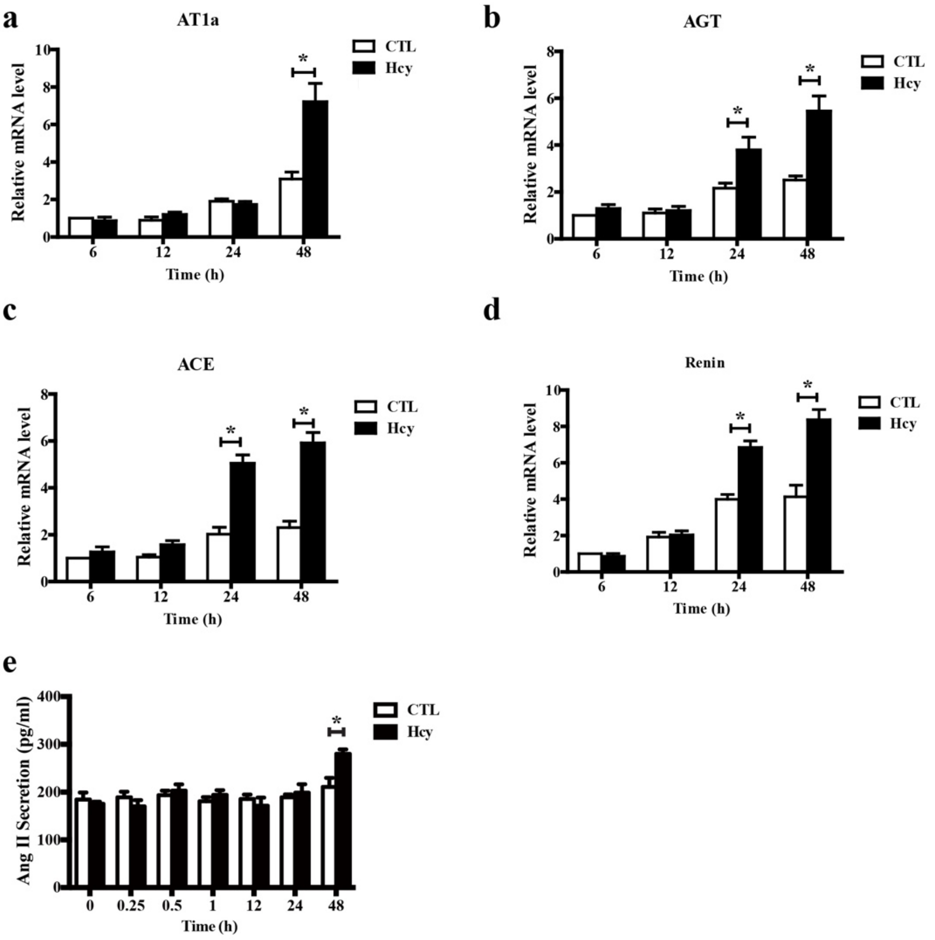

Supplementary Figure 5. Hcy does not upregulate RAAS expression until 24 to 48 hours and does not affect Ang II production. a-d, Hcy-induced mice abdominal aortic ring AT1a, AGT, ACE and renin mRNA transcription at different time points. The data represent as mean ± SEM, N=6, *P<0.05, Student's t-test. **e,** Hcy-induced mice abdominal aortic ring Ang II secretion at different time points. The data represent as mean ± SEM, N=6, *P<0.05, Student's t-test.

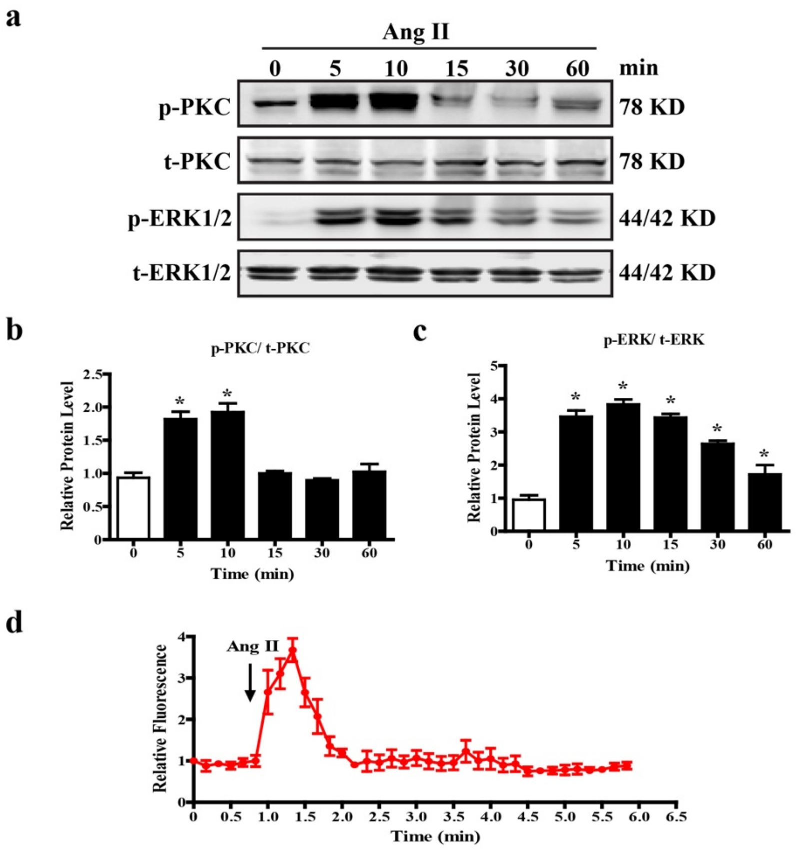

Supplementary Figure 6. Ang II activates AT1 receptor G_q signaling. a-c, Representative Western blots and quantification of phosphorylated and total PKC and ERK1/2 in Ang II (1 μM)-treated HEK293A cells (transfected with the human AT1 receptor). The data represent as mean ± SEM, N=6, *P<0.05, One-way ANOVA followed by the Bonferroni post hoc test **d,** Ca^{2+} signaling in HEK293A cells (transfected with the human AT1 receptor) stimulated with Ang II (1 μM). The data represent as mean ± SEM, N=6.

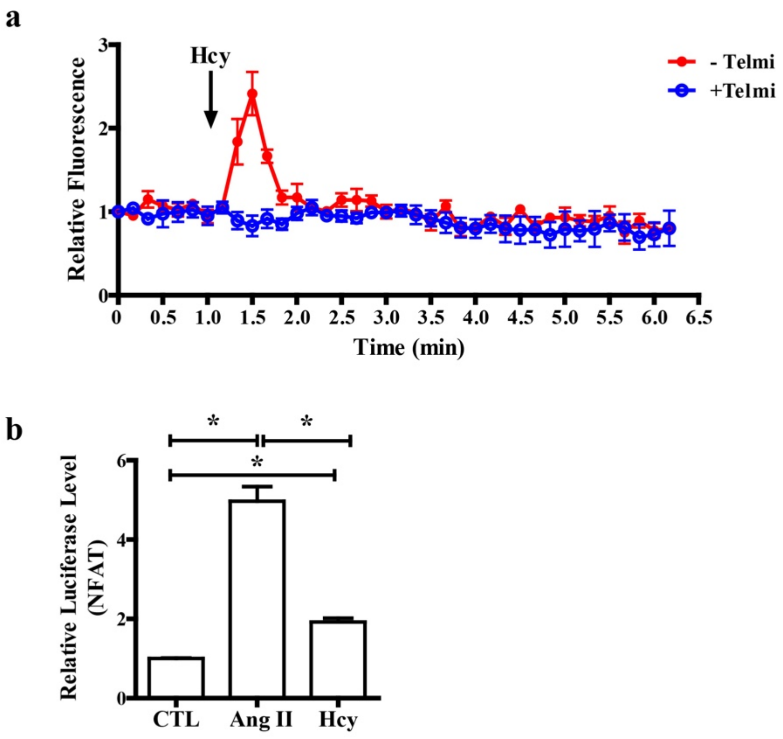

Supplementary Figure 7. Hcy induces Ca^{2+} and NFAT signaling. a, Ca^{2+} signaling in HEK293A cells (transfected with the human AT1 receptor) stimulated with Hcy (100 μM) with or without telmisartan (1 μM). The data represent as mean ± SEM, N=6. **b,** Hcy (100 μM) and Ang II (1 μM)-induced NFAT signaling. The data represent as mean ± SEM, N=6, *P<0.05, One-way ANOVA followed by the Bonferroni post hoc test.

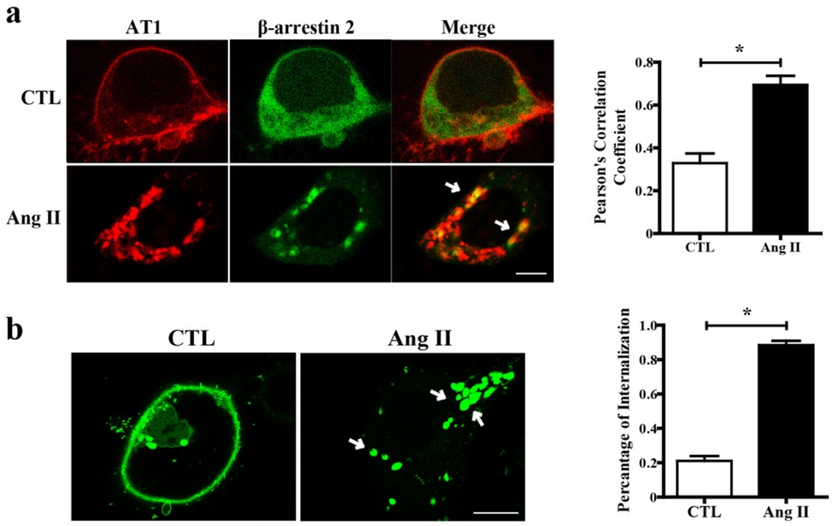

Supplementary Figure 8. Ang II activates AT1 receptor β-arrestin 2 signaling. a, Representative fluorescence and quantification of Ang II (1 μM)-induced colocalization of the AT1 receptor (human AT1 receptor labeled with mCherry; red) with β-arrestin 2 (β-arrestin 2 labeled with GFP; green) in cultured HEK293A cells. The merged area is indicated as yellow (arrows). Scale bar, 10 μm. The data represent as mean ± SEM, N=6, *P<0.05, Mann-Whitney test. **b,** Representative fluorescence and quantification of Ang II (1 μM)-induced AT1 receptor (mouse AT1a receptor labeled with GFP; green) internalization (arrows) in COS7 cells. The data represent as mean ± SEM, N=10, *P<0.05, Mann-Whitney test.

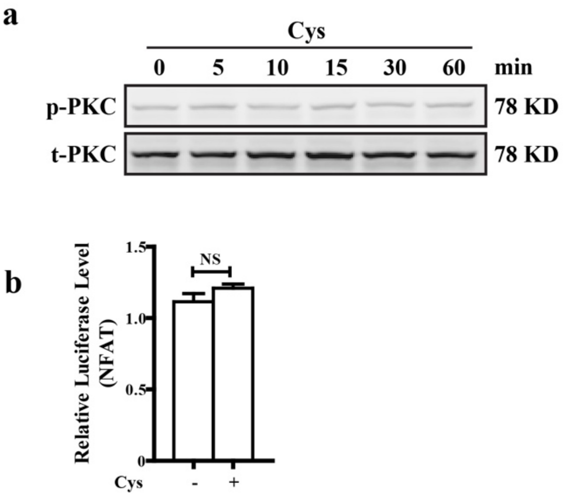

Supplementary Figure 9. Cysteine does not activate the AT1 receptor. a, Representative Western blots of phosphorylated and total PKC in cysteine (100 μM)-treated HEK293A cells (transfected with the human AT1 receptor). N=6. **b,** NFAT signaling of HEK293A cells (transfected with the human AT1 receptor) after cysteine (100 μM) treatment, detected by the dual luciferase assay system (Promega). The data represent as mean ± SEM, N=6. NS, no significance, Student's t-test.

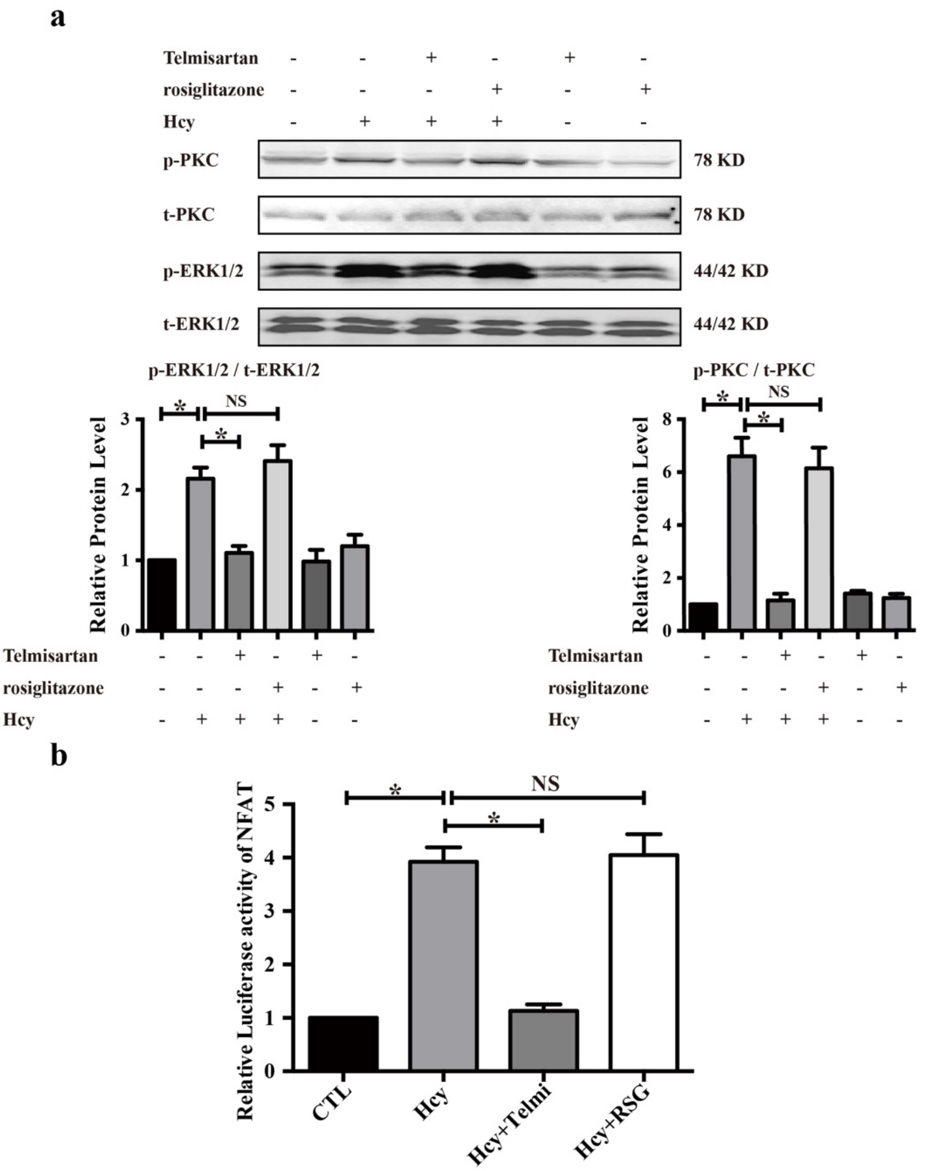

Supplementary Figure 10. Sartans inhibited Hcy-induced AT1 activation independent on its PPARγ-activating effect. a, Representative Western blots and quantification of phosphorylated and total PKC and ERK1/2 in Hcy (100 μM)-treated HEK293A cells (transfected with the human AT1 receptor) with or without telmisartan (1 μM) or rosiglitazone (20 μM). The data represent as mean ± SEM, N=6. *P<0.05. One-way ANOVA followed by the Bonferroni post hoc test. **b,** Hcy (100 μM) -induced NFAT signaling with or without telmisartan (1 μM) and rosiglitazone (20 μM). The data represent as mean ± SEM, N=6. *P<0.05, NS, no significance. One-way ANOVA followed by the Bonferroni post hoc test.

c

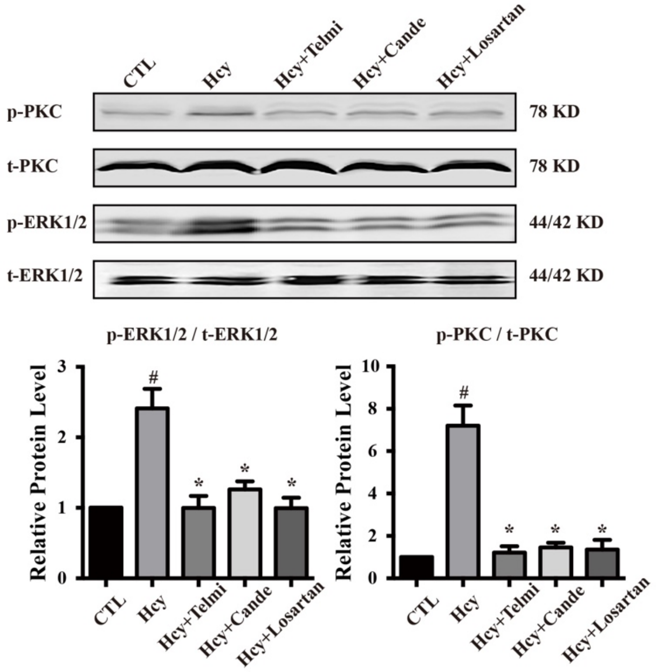

d

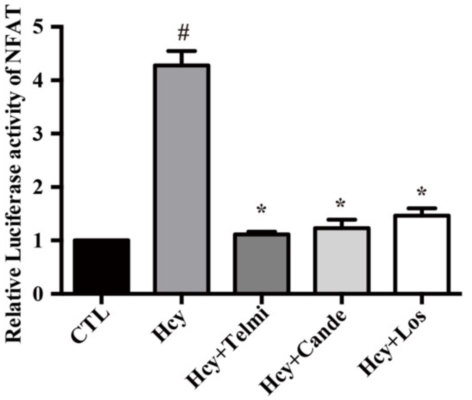

Supplementary Figure 10. Sartans inhibited Hcy-induced AT1 activation independent on its PPARγ-activating effect. c, Representative Western blots and quantification of phosphorylated and total PKC and ERK1/2 in Hcy (100 μM)-treated HEK293A cells (transfected with the human AT1 receptor) with telmisartan (1 μM), candesartan (1 μM) or losartan (1 μM). The data represent as mean ± SEM, N=6. $^{\#}P$<0.05 *vs*. CTL. Hcy. *P<0.05 *vs*. Hcy. One-way ANOVA followed by the Bonferroni post hoc test. **d,** Hcy (100 μM) -induced NFAT signaling with telmisartan (1 μM), candesartan (1 μM) or losartan (1 μM). The data represent as mean ± SEM, N=6. $^{\#}P$<0.05 *vs*. CTL. Hcy. *P<0.05 *vs*. Hcy. One-way ANOVA followed by the Bonferroni post hoc test.

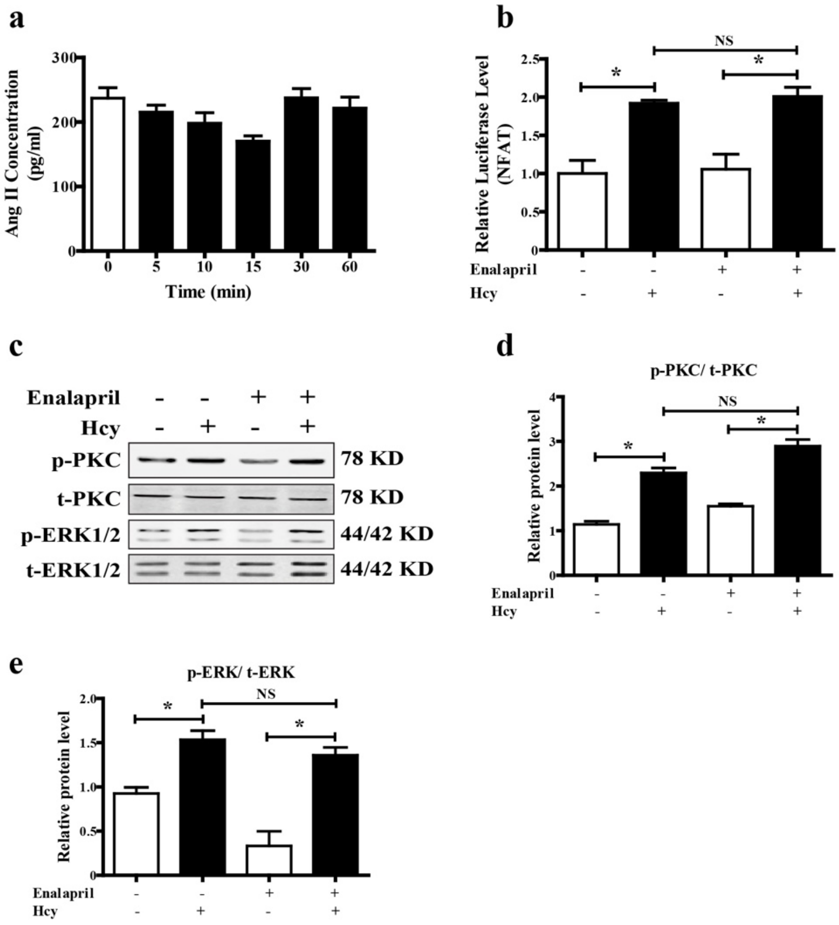

Supplementary Figure 11. Hcy-induced AT1 receptor activation is not due to Ang II production. a, Ang II concentration in Hcy (100 μM)-treated HEK293A cells transfected with the human AT1 receptor. N=6. One-way ANOVA followed by the Bonferroni post hoc test. **b,** Hcy (100 μM)-induced NFAT signaling with or without enalapril (1 μM) pretreatment in HEK293A cells expressing the human AT1 receptor, detected by the dual luciferase assay system (Promega). The data represent as mean ± SEM, N=6. *$P<0.05$. Two-way ANOVA followed by the Bonferroni post hoc test. **c-e,** Representative Western blots and quantification of phosphorylated and total PKC and ERK1/2 in Hcy (100 μM)-treated HEK293A cells (transfected with the human AT1 receptor) with or without enalapril (1 μM) pretreatment. The data represent as mean ± SEM, N=6, *$P<0.05$. Two-way ANOVA followed by the Bonferroni post hoc test.

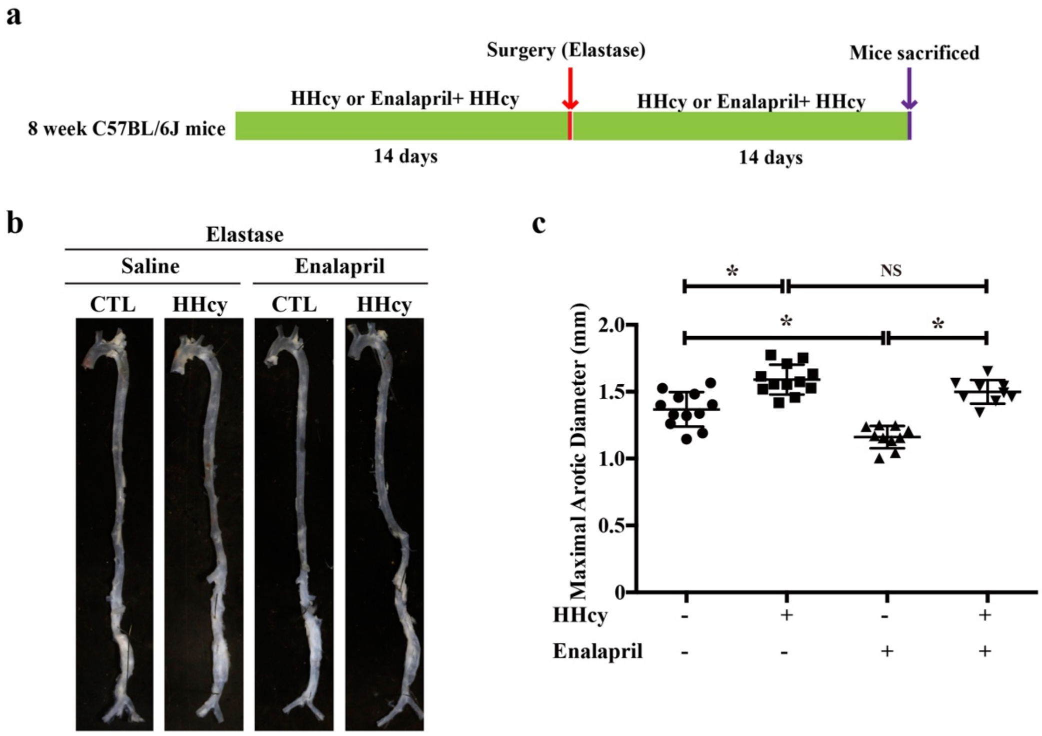

Supplementary Figure 12. Enalapril treatment does not alleviate HHcy-aggravated AAA in mice. a, Schematic flowcharts of animal models with HHcy induction, enalapril feeding or elastase treatment. **b,** Representative photographs of elastase-induced AAA in C57BL/6J mice fed with or without enalapril and Hcy. N=9-12. **c,** The quantification of the infrarenal abdominal aortic diameter in mice with elastase-induced aneurysms. The data represent as mean ± SEM, N=9-12. *P<0.05. NS, no significance. Kruskal-Wallis test followed by Dunn's test.

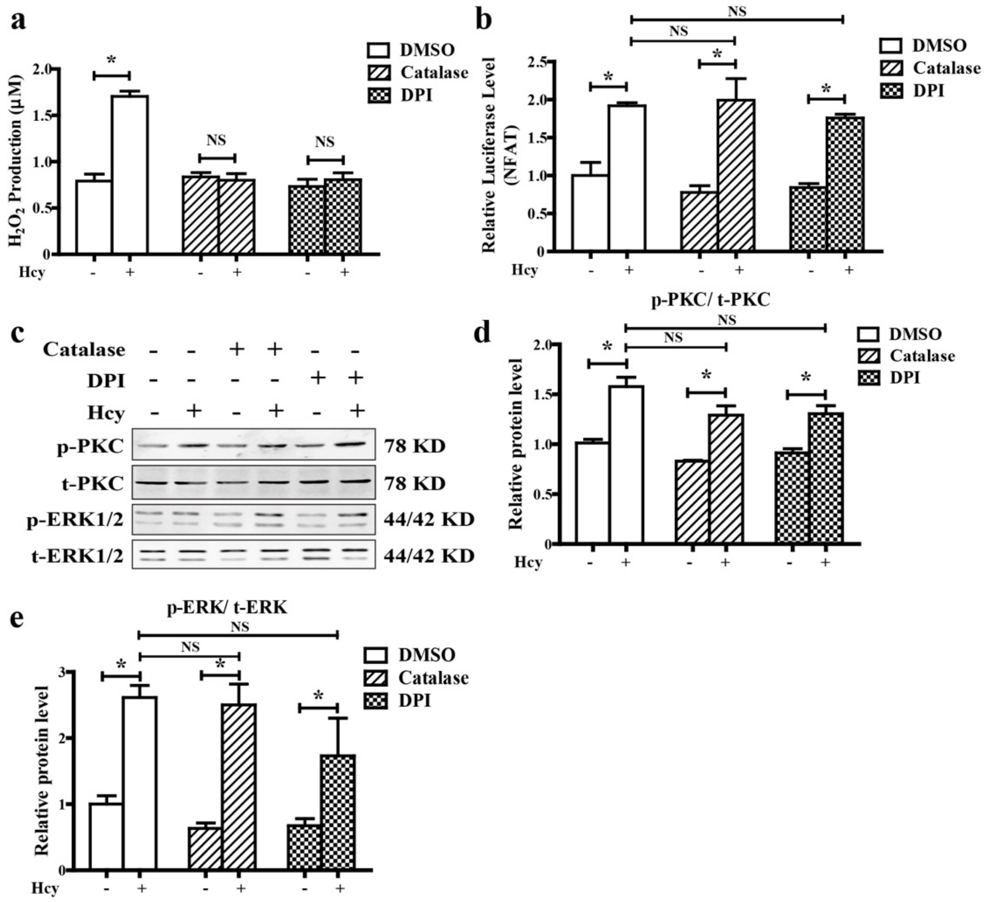

Supplementary Figure 13. Hcy-induced AT1 receptor activation is not due to ROS production. a, Hcy-induced H_2O_2 production in HEK293A cells with or without the H_2O_2 scavenger catalase (2000 U) or diphenyliodonium (DPI, 10 μM) pretreatment was detected by Amplex Red. The data represent as mean ± SEM, N=6. *$P<0.05$. NS, no significance. Two-way ANOVA followed by the Bonferroni post hoc test. **b,** Hcy (100 μM)-induced NFAT signaling with or without the H_2O_2 scavenger catalase (2000 U) or DPI (10 μM) pretreatment in HEK293A cells expressing the human AT1 receptor, detected by the dual luciferase assay system (Promega). The data represent as mean ± SEM, N=6. *$P<0.05$. NS, no significance. Two-way ANOVA followed by the Bonferroni post hoc test. **c-e,** Representative Western blots and quantification of phosphorylated and total PKC and ERK1/2 in Hcy (100 μM)-treated HEK293A cells (transfected with the human AT1 receptor) with or without the H_2O_2 scavenger catalase (2000 U) or DPI (10 μM) pretreatment. The data represent as mean ± SEM, N=6. *$P<0.05$. NS, no significance. Two-way ANOVA followed by the Bonferroni post hoc test.

a

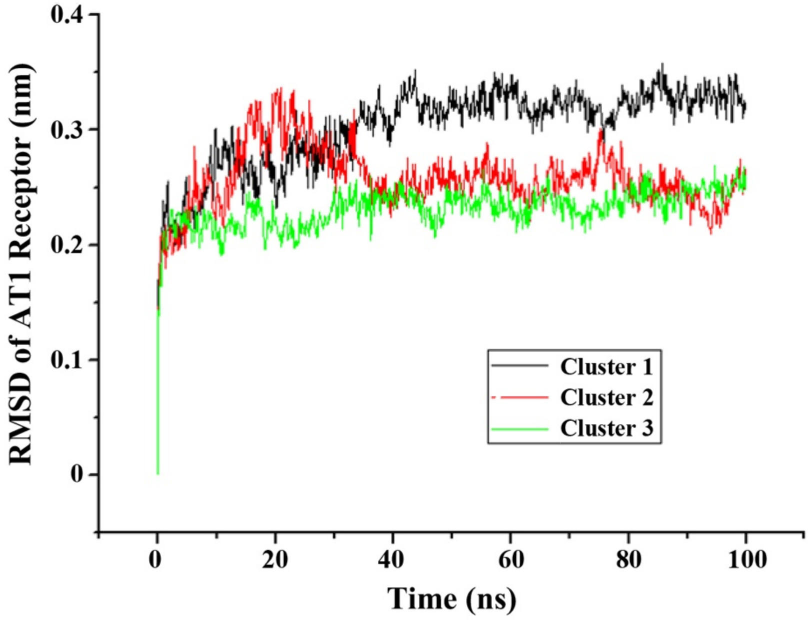

b

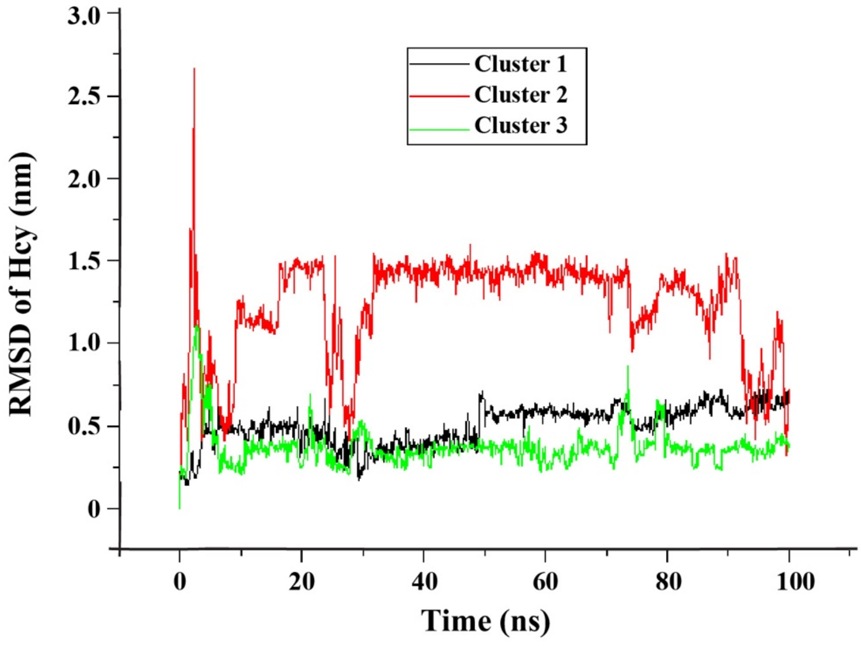

Supplementary Figure 14. RMSD of the AT1 receptor (a) and Hcy (b) in Cluster 1 to Cluster 3.

a

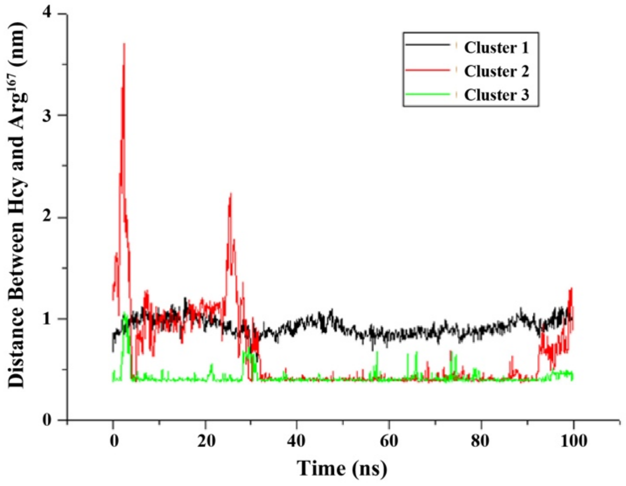

b

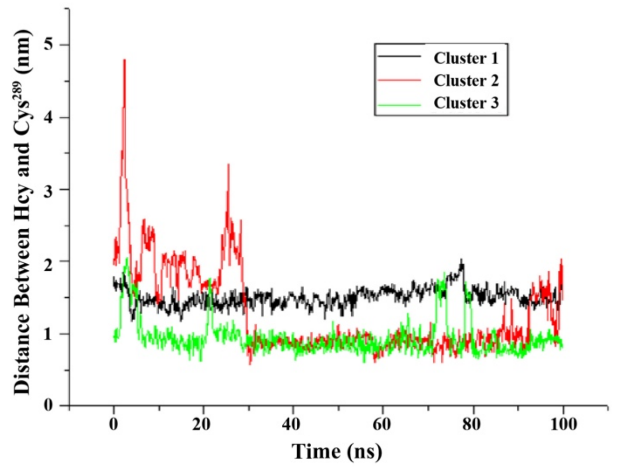

Supplementary Figure 15. Distance between Hcy and Arg^{167} (a) or Cys^{289} (b) of the AT1 receptor.

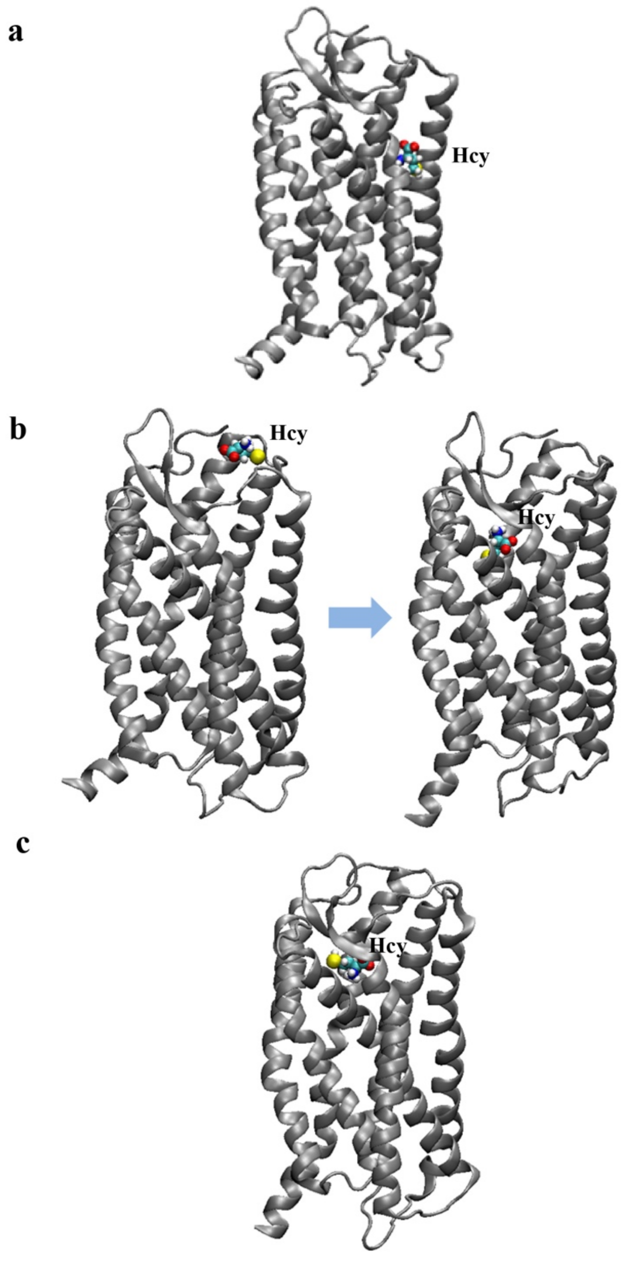

Supplementary Figure 16. Locations of Hcy in Cluster 1 (a), Cluster 2 (b) and Cluster 3 (c).

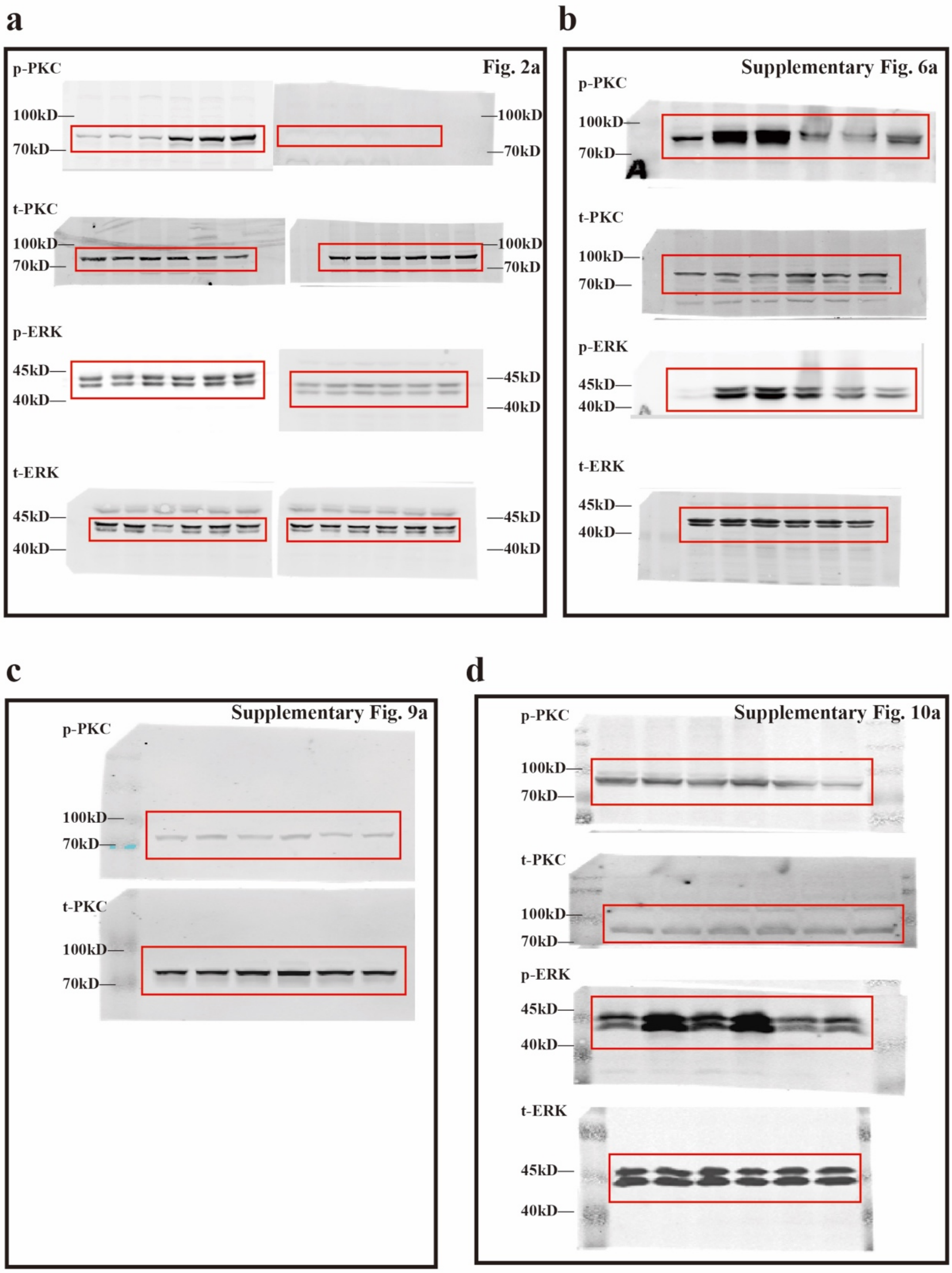
a
Fig. 2a
p-PKC
100kD
70kD
100kD
70kD
t-PKC
100kD
70kD
100kD
70kD
p-ERK
45kD
40kD
45kD
40kD
t-ERK
45kD
40kD
45kD
40kD
b
Supplementary Fig. 6a
p-PKC
100kD
70kD
t-PKC
100kD
70kD
p-ERK
45kD
40kD
t-ERK
45kD
40kD
c
Supplementary Fig. 9a
p-PKC
100kD
70kD
t-PKC
100kD
70kD
d
Supplementary Fig. 10a
p-PKC
100kD
70kD
t-PKC
100kD
70kD
p-ERK
45kD
40kD
t-ERK
45kD
40kD

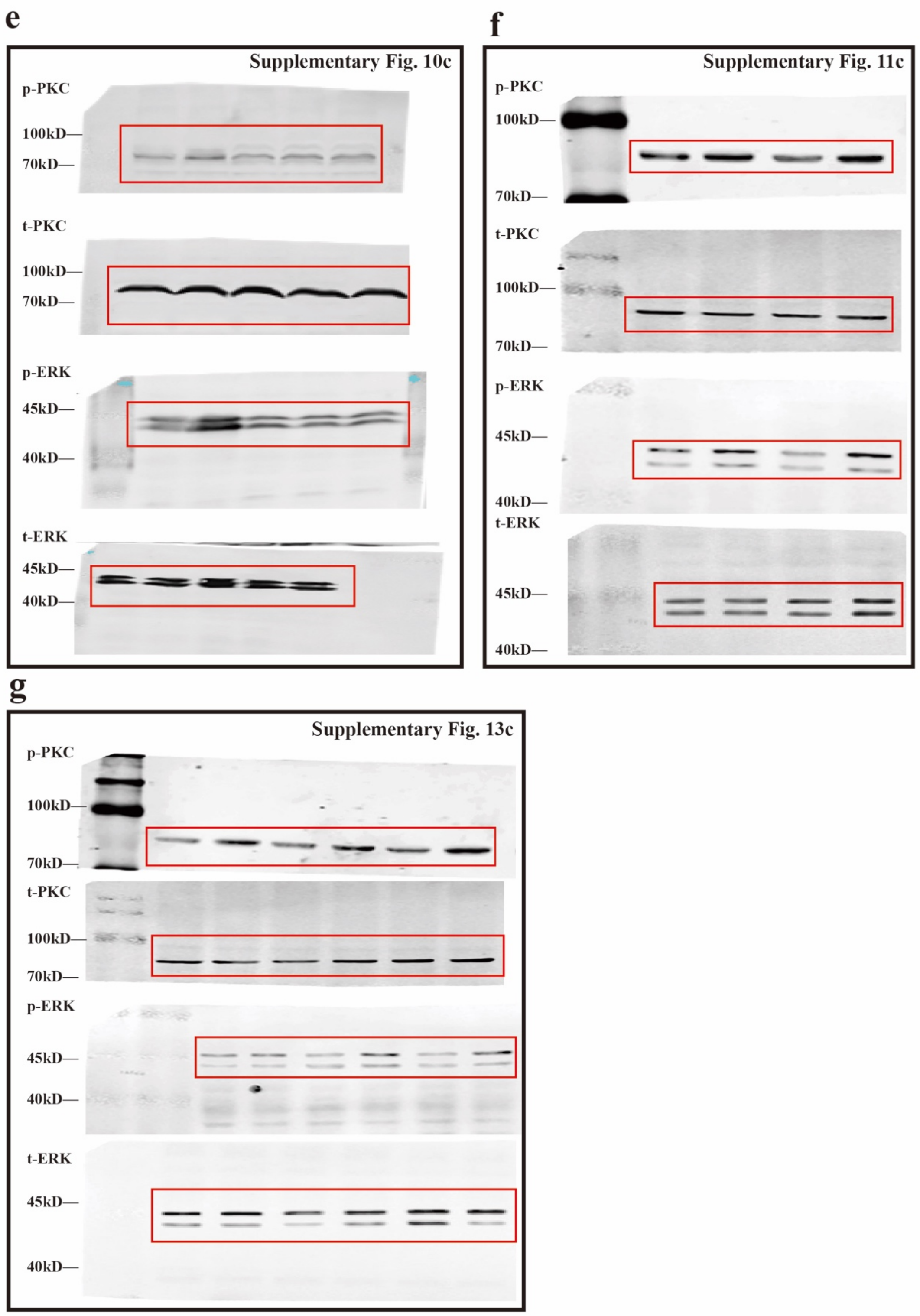

Supplementary Figure 17. Full gel scans for western blot. a, Gel scans for Fig. 2a. **b,** Gel scans for Supplementary Fig. 6a. **c,** Gel scans for Supplementary Fig. 9a. **d,** Gel scans for Supplementary Fig. 10a. **e,** Gel scans for Supplementary Fig. 10c. **f,** Gel scans for Supplementary Fig. 11c. **g,** Gel scans for Supplementary Fig. 13c.

Supplementary Table 1. Characteristics of WT and *AT1a*$^{-/-}$ mice in elastase-induced AAA model fed with or without Hcy.

Group	sham	Elastase		sham	Elastase	
	WT	WT	WT HHcy	*AT1a*$^{-/-}$	*AT1a*$^{-/-}$	*AT1a*$^{-/-}$ HHcy
No.	5	12	12	5	12	9
Weight (g)	25.2±1.5	23.1 ± 0.7	24.5 ± 0.8	23.6±1.6	23.6 ± 0.5	25.0 ± 0.7
SBP before (mmHg)	105.4±1.8	108.4 ± 4.3	109.2 ± 3.7	74.1±3	74.3 ± 1.4*	73.6 ± 2.3*
SBP after (mmHg)	105.9±2.4	109.7 ± 2.4	107.3 ± 2.9	73.9±3.6	70.3 ± 1.2*	71.9 ± 2.0*
Plasma Ang II (pg/ml)	455.9±15.3	448.3 ± 12.76	470.3 ± 15.06	555.2±14.1	522.6±18.73*	536.8 ± 9.64*
Plasma Total Hcy (μM)	7.47±1.53	8.44 ± 0.75	25.28 ± 2.13†	6.34±0.53	8.10 ± 0.50	23.60 ± 2.70†
Plasma Free Hcy (μM)	0.29±0.04	0.28±0.01	0.58±0.02†	0.27±0.03	0.21±0.01	0.68±0.09†
TC (mM)	2.02±0.11	2.17 ± 0.09	2.12 ± 0.12	1.96±0.13	1.92 ± 0.06	2.02 ± 0.11
TG (mM)	0.96±0.07	0.97 ± 0.08	1.14 ± 0.10	0.96±0.03	1.01 ± 0.05	1.07 ± 0.10

WT, wild type; HHcy, hyperhomocysteinemia; *AT1a*$^{-/-}$, AT1a knockout; SBP, systolic blood pressure; Ang II, angiotensin II; Hcy, homocysteine; TC, total cholesterol; TG, triglyceride.

Data represent mean ± SEM. **P*<0.05 compared to WT or WT HHcy mice with elastase induction accordingly. †*P*<0.05 compared to elastase-induced WT or *AT1a*$^{-/-}$ mice fed without Hcy accrodingly.

Supplementary Table 2. Characteristics of WT and *AT1a*$^{-/-}$ mice in CaPO$_4$-induced AAA model fed with or without Hcy and telmisartan.

Group	WT	WT HHcy	*AT1a*$^{-/-}$	*AT1a*$^{-/-}$ HHcy
No.	9	9	12	5
Weight (g)	26.6 ± 1.7	26.2 ± 0.8	26.9 ± 0.9	26.3 ± 1.2
SBP before (mmHg)	102.8 ± 1.8	101.7 ± 2.1	72.5 ± 6.2*	72.9 ± 2.5*
SBP after (mmHg)	104.7 ± 1.9	104.9 ± 3.5	68.7 ± 2.0*	71.9 ± 1.8*
Plasma Ang II (pg/ml)	437.9 ± 12.23	437.9 ± 16.48	539.9 ± 25.62*	524.9 ± 25.35*
Plasma Total Hcy (μM)	7.84 ± 0.93	20.55 ± 3.21†	9.40 ± 1.80	18.24 ± 3.10†
Plasma Free Hcy (μM)	0.29±0.08	0.61±0.07†	0.27±0.04	0.59±0.11†
TC (mM)	1.87 ± 0.13	1.80 ± 0.04	1.98 ± 0.20	1.91 ± 0.20
TG (mM)	1.02 ± 0.28	1.16 ± 0.10	1.17 ± 0.43	1.26 ± 0.20

WT, wild type; HHcy, hyperhomocysteinemia; *AT1a*$^{-/-}$, AT1a knockout; SBP, systolic blood pressure; Ang II, angiotensin II; Hcy, homocysteine; TC, total cholesterol; TG, triglyceride.

Data represent mean ± SEM. *P<0.05 compared to WT or WT HHcy mice accordingly. †P<0.05 compared to WT or *AT1a*$^{-/-}$ mice fed without Hcy accordingly.

Supplementary Table 3. Characteristics of C57BL/6J mice in $CaPO_4$-induced AAA model fed with or without Hcy and telmisartan.

Group	CTL	HHcy	Telmi	Telmi + HHcy
No.	9	9	8	7
Weight (g)	24.8 ± 0.5	23.3 ± 0.5	22.9 ± 0.6	22.7 ± 0.6
SBP before (mmHg)	101.3 ± 2.1	103.9 ± 2.2	102.6 ± 2.7	106.0 ± 2.0
SBP after (mmHg)	98.0 ± 3.4	106.6 ± 3.5	96.7 ± 4.2	96.8 ± 3.4
Plasma Ang II (pg/ml)	442.8 ± 20.27	435.1 ± 20.27	538.2 ± 19.69*	521.5 ± 16.95*
Plasma Total Hcy (μM)	8.02 ± 0.73	25.75 ± 2.89†	10.64 ± 1.35	22.62 ± 2.94†
TC (mM)	2.12 ± 0.10	2.12 ± 0.11	2.10 ± 0.04	2.09 ± 0.15
TG (mM)	0.72 ± 0.03	0.61 ± 0.03	0.77 ± 0.07	0.72 ± 0.08

CTL, control; HHcy, hyperhomocysteinemia; Telmi, telmisartan; SBP, systolic blood pressure; Ang II, angiotensin II; Hcy, homocysteine; TC, total cholesterol; TG, triglyceride.

Data represent mean ± SEM. *$P<0.05$ compared to CTL or HHcy mice fed without telmisartan accordingly. †$P<0.05$ compared to CTL or Telmi mice fed without Hcy accordingly.

Supplementary Table 4. Impact of Telmi (1 μM) upon Hcy-induced Ca^{2+} signaling.

	-Log [EC_{50} (M)]	Maximum Responses (%)
Hcy	4.09 ± 0.39	96.4 ± 11.9
Telmi + Hcy	4.77 ± 1.37	23.8 ± 4.38*

The data represent the mean ± SEM from 3 independent experiments performed in duplicate or triplicate. *p<0.05.

Supplementary Table 5. Impact of Telmi (1 µM) upon Hcy-induced NFAT signaling.

	-Log [EC_{50} (M)]	Maximum Responses (%)
Hcy	5.26 ± 0.11	104 ± 3.16
Telmi + Hcy	4.70 ± 0.62	16.5 ± 2.58*

The data represent the mean ± SEM from 3 independent experiments performed in duplicate or triplicate. *p<0.05.

Supplementary Table 6. Characteristics of C57BL/6J mice in Elastase-induced AAA model fed with or without Hcy and enalapril.

Group	CTL	HHcy	Enalapril	Enalapril +HHcy
No.	11	9	11	9
Weight (g)	26.2±0.3	24.4±0.9	25.0±0.6	26.9±0.8
SBP before (mmHg)	101.2±1.6	105.2±2.3	102.3±3.4	99.7±2.9
SBP after (mmHg)	107.8±1.4	109.1±1.6	84±1.3*	82.44±1.6*
Plasma Total Hcy (μM)	6.17±0.46	25.24±2.48†	5.83±0.52	25.38±1.19†
Plasma Ang II (pg/ml)	450.1±19.5	489.5±29.8	270.6±13.3*	295.6±13.6*
Plasma Renin Activity (ng Ang I/ml.hr)	3.21±0.43	3.01±0.24	36.54±3.50*	35.99±2.09*

HHcy, hyperhomocysteinemia; SBP, systolic blood pressure; Hcy, homocysteine; Data represent mean ± SEM. **P*<0.05 compared to CTL or HHcy mice fed without enalapril accordingly. †*P*<0.05 compared to CTL or enalapril mice fed without Hcy accordingly.

Supplementary Table 7. Impact of Hcy (100 µM) upon Ang II-induced Ca^{2+} signaling.

	-Log [EC_{50} (M)]	Maximum Responses (%)
Ang II	11.8 ± 0.18	99.7 ± 5.03
Ang II + Hcy	10.7 ± 0.23*	152 ± 10.3*

The data represent the mean ± SEM from 3 independent experiments performed in duplicate or triplicate. *p<0.05.

Supplementary Table 8. Impact of Hcy upon Ang II-induced NFAT signaling.

	-Log [EC_{50} (M)]	Maximum Responses (%)
Ang II	7.10 ± 0.22	98.22 ± 5.67
Ang II + Hcy (30 μM)	7.39 ± 0.08	109.4 ± 2.14
Ang II + Hcy (60 μM)	7.39 ± 0.18	119.6 ± 4.98*
Ang II + Hcy (100 μM)	7.61 ± 0.14	152.7 ± 4.57*

The data represent the mean ± SEM from 3 independent experiments performed in duplicate or triplicate. *****p<0.05.

Supplementary Table 9. Primer sequences for real time PCR.

Gene	Primer	Primer Sequence
Mouse AT1a	Sense	5'-GCCCTGGCTGACTTATGCTT-3'
	Antisense	5'-ACACATTTCGGTGGATGACGG-3'
Mouse AGT	Sense	5'-ATCCCTTAAACTTTCACAACC-3'
	Antisense	5'-CGGAACTTCTAGCACACC-3'
Mouse ACE	Sense	5'-ATTTGGCAGAACTTTACTGAC-3'
	Antisense	5'-CAAACAACAACTTGGCATAG-3'
Mouse Renin	Sense	5'- CCACCTTCATCCGCAAGTTC-3'
	Antisense	5'- GGGCAACACTCGTTAGGGTCT-3'
Mouse β-actin	Sense	5'-ATCTGGCACCACACCTTC-3'
	Antisense	5'-AGCCAGGTCCAGACGCA-3'
